# Enzymatic Time-Temperature Indicator Prototype Developed by Immobilizing Laccase on Electrospun Fibers to Predict Lactic Acid Bacterial Growth in Milk during Storage

**DOI:** 10.3390/nano11051160

**Published:** 2021-04-29

**Authors:** Ting-Yu Tsai, Shih-Hsin Chen, Li-Chen Chen, Shih-Bin Lin, Shyi-Neng Lou, Yen-Hui Chen, Hui-Huang Chen

**Affiliations:** 1 Department of Food Science, National Ilan University, 1, Sec. 1, Shennong Rd., Yilan County, Yilan City 26047, Taiwan; ging580360@gmail.com (T.-Y.T.); ywchen@niu.edu.tw (L.-C.C.); sblin@niu.edu.tw (S.-B.L.); snlou@niu.edu.tw (S.-N.L.); yenhui@niu.edu.tw (Y.-H.C.); 2 Institute of Food Science and Technology, National Taiwan University, 1, Sec. 4, Roosevelt Rd., Taipei 10617, Taiwan; shchen0422@ntu.edu.tw

**Keywords:** TTI, electrospinning, laccase, milk, lactic acid bacteria

## Abstract

Laccase was immobilized on a chitosan/polyvinyl alcohol/tetraethylorthosilicate electrospun film (ceCPTL) and colored with guaiacol to obtain a laccase time–temperature indicator (TTI) prototype. The activation energy (*Ea*) of coloration of the prototype was 50.89–33.62 kJ/mol when 8–25 μg/cm^2^ laccase was immobilized on ceCPTL, and that of lactic acid bacteria (LAB) growth in milk was 73.32 kJ/mol. The *Ea* of coloration of the TTI prototype onto which 8–10 μg/cm^2^ laccase was immobilized was in the required range for predicting LAB growth in milk. The coloration endpoint of the TTI prototype onto which 10 μg/cm^2^ (0.01 U) laccase was immobilized could respond to the LAB count reaching 10^6^ colony-forming units (CFU)/mL in milk during a static temperature response test, and the prediction error was discovered to be low. In dynamic temperature response experiments with intermittent temperature changes between 4 and 25 °C, the coloration rate of the laccase TTI prototype was consistent with LAB growth. The results of this study indicate that the laccase TTI prototype can be applied as a visual monitoring indicator to assist in evaluating milk quality in cold chains.

## 1. Introduction

The function of packaging is not only to hold food. In recent years, package has expanded its function to protect food and improve storage quality (active packaging), to offer the quality information on the package (intelligent packaging), and to offer the communication function of the package to facilitate communication with consumers throughout the supply chain (smart packaging). Therefore, an intelligent food package should not only serve the purposes of containment and protection but also provide consumers with essential information regarding food quality to ensure food safety. Temperature fluctuation in the storage environment is the main cause of unpredictable food spoilage, and large temperature fluctuations may considerably influence the quality of food throughout its life cycle [1].

As an intelligent food packaging technology, a time–temperature indicator (TTI) enables monitoring food quality by its color changes based on the accumulation information of temperature and time, and it can be used to predict the condition of packaged food or its environment [2,3]. A TTI provides a continuous time–temperature history of foods, thus providing managers and consumers with reliable and accurate information related to food quality and safety. The TTI device is attached to the outer packaging of food and records and provides the time and temperature history of a food to observers [2].

The functions of TTIs are based on physical, chemical, microbial, or biological mechanisms, such as diffusion emigration, monomer polymerization, microbial reactions, and enzymatic reactions, respectively [3]. An enzymatic TTI is generally designed to undergo catalysis for exhibiting coloration, is more sensitive than other TTIs to environmental temperature changes, and is more accurate than a chemical or physical-diffusion-based TTI [4]. Therefore, enzymatic TTI systems have been extensively researched. Laccases, which are multicopper oxidases, are common natural enzymes that can be used in biosensors. In addition to undergoing a simple catalytic reaction, laccases can react with various substrates, including amines and aromatic compounds [5]. Laccase enzymes withdraw electrons from substrates, converting the substrates into free radicals, which can be polymerized to develop pigments [6]. Fungal-derived laccases—which catalyze the oxidation of phenolic compounds such as aminophenols, polyphenols, and phenols—can oxidize guaiacol; this reaction is observed as a color change from transparent to deep brown or deep purplish brown [5,7].

Laccases are safe and ecofriendly and have recently received considerable attention as promising candidates for optical biosensing applications [5,8]. However, enzymatic TTIs have some limitations, including enzyme instability and irreversible deactivation when temperature and storage duration are high [9]. Moreover, enzymes are expensive. The commercially available TTI is about 2–4 US dollars per piece. Such a price is still too high to be applicable to individual products, which means the TTI cannot be widely used in the food industry at present. Enzyme stability during storage should be improved and monitored, and the amount of enzyme required should be reduced to increase cost-effectiveness; these actions would enable the development of commercial enzymatic TTI systems. Immobilization is one of the preferred methods for improving enzyme stability and is achieved by steading enzyme structure to resist changes in environmental temperature, pH, etc., and would reduce the inhibition of reaction products in the electrospun polyacrylonitrile nanofibrous membranes [10]. Among the various approaches used for enzyme immobilization, electrospinning is a progressive and desirable method that produces fibers (with diameter ranging from submicrons to several nanometers) in a high-voltage electrostatic field to obtain a material with a large specific surface area; this area is then available for laccase immobilization with exceptional stability [7,10,11]. Moreover, a large void between immobilized enzymes on such nanofibers results in fewer spatial structural obstacles and thus leads to contact and reactions between enzymes and reactants [12,13]. Chitosan (CS) is an excellent biomaterial for use in enzyme immobilization [7]. However, electrospinning of CS has some limitations; for example, creating continuous fiber jets during electrospinning is difficult because of the repulsive forces between ionic groups and the high surface tension of the polymer solution given that CS has high viscosity even at moderate concentrations. To overcome these limitations, CS is commonly blended with other polymers for electrospinning, such as polyvinyl alcohol (PVA) [14].

Tetraethyl orthosilicate tetraethylorthosilicate (TEOS) has been used to increase the adhesion of electrospun fibers and the stability of electrospun CS fiber films [15]. In the present study, laccase was immobilized on CS/PVA/TEOS electrospun fibers to develop a laccase TTI prototype for simultaneously reducing the amount and stabilizing the activity of an immobilized enzyme.

Milk is a perishable food because it is an ideal medium for the growth of different microorganisms, which leads to its early deterioration. In a cold chain, low-temperature storage is necessary at every stage for maintaining pasteurized milk quality and safety. The US Food and Drug Administration (FDA) advises consumers to be aware of the temperature and duration of storage when consuming this high-risk food [16] because milk may deteriorate or even spoil before its expiration date if temperature changes occur during transportation and storage in the cold chain. The shelf life printed on food packages is not sufficiently reliable to ensure food quality and safety. The monitoring of temperature fluctuations during food transportation and storage is crucial for determining food quality. Although lactic acid bacteria (LAB) may not be the main spoilage bacteria causing milk deterioration, they are commonly found in expired pasteurized milk in Taiwan and may decrease the pH of dairy products by metabolizing lactose into lactic acid [17]. In the present study, a biosensor capable of displaying color changes was developed to respond to LAB growth and to visually predict milk quality. The TTI developed in this study is attached to the outer packaging of milk and is expected to be used as an intelligent packaging. If a TTI is employed to predict food quality or shelf life, overestimation of quality is unacceptable. To prevent such overestimation, Arrhenius activation energy (*Ea*) was estimated to establish guidelines for temperature dependency, and response tests of a laccase TTI prototype designed in this study were conducted for predicting LAB growth in milk during temperature fluctuations.

## 2. Material and Methods

### 2.1. Immobilization of Laccase on Electrospun Fibers

Following the method of Pirzada et al. [15], a composite gel solution was blended with PVA (118–124 kDa, First Chemical Manufacture Co., Ltd., Taipei, Taiwan), CS (deacetylation degree: 76%, 50–190 kDa, Sigma-Aldrich, St. Louis, MO, USA), and TEOS (Sigma-Aldrich) for electrospinning. To increase the ability of the electrospun fibers to adhere to the polypropylene (PP) film, approximately 2 g of TEOS was dissolved in 3 mL of acetic acid solution (1 mol/L) in a round-bottom flask. Subsequently, 4 mL of 10 wt% PVA and 6 mL of 3 wt% CS/acetic acid (1 mol/L) were added to the aforementioned solution, which was stirred at 100 rpm for 50 min in a water bath at 60 °C. Finally, a homogeneous CS/PVA/TEOS gel solution was obtained. Ultrasonic degassing was performed for 5 min in the water bath. The CS/PVA/TEOS gel solution was then fed into a positively charged spinneret attached to an electrospinning apparatus (NE-300, Falco Enterprise, New Taipei City, Taiwan) operated at 15 kV. The gel solution used in electrospinning was placed in a 5-mL syringe with a No. 21 needle and was then injected out at a feed rate of 0.5 mL/h. A PP film was attached to a drum collector. The rotating speed of the drum collector was 100 rpm and was placed at a distance of 20 cm from the needle tip to collect the electrospun fibers. The completed single-side electrospun film (CS/PVA/TEOS/PP) was soaked in 3% glutaraldehyde (GA; Nihon Shiyaku Industries Ltd., Osaka, Japan) and was incubated for 2 h to covalently bond an aldehyde group of GA to an amine group on the electrospun CS/PVA/TEOS fiber. Subsequently, the GA-modified CS/PVA/TEOS was washed with acetate buffer (pH 4.5) and dried overnight in a desiccator at ambient temperature to produce CS/PVA/TEOS/PP/GA. Three sheets of CS/PVA/TEOS/PP/GA were generated, and 20 pieces of 1 cm^2^ were cut from the middle of each sheet. A minimum of three pieces were randomly selected for enzyme immobilization and coloration tests. In enzyme immobilization, 50 μL of 0.16–0.5 μg/μL laccase solution (Sigma-Aldrich, from *Trametes versicolor*, lot result: 1.07 U/mg) was spread carefully on a 1-cm^2^ piece of CS/PVA/TEOS/PP/GA to immobilize 8–25 μg (8.56–26.75 × 10^−3^ U) of laccase on each piece of film. The process of laccase immobilization by crosslinking involves binding an aldehyde group of GA to an amine group on chitosan, followed by the binding of an amino group of laccase with another aldehyde group, which has been proved according to Fourier-transform infrared spectroscopy analysis by Jhuang et al. [7]. The enzyme-immobilized electrospun film was dried in an oven for 1 h at ambient temperature to produce CS/PVA/TEOS/PP/GA/laccase (ceCPTL), which was stored at 4 °C. In addition, the occurrence of a coupling reaction in ceCPTL was tested by rinsing ceCPTL and then the rinsing liquid was reacted with a guaiacol solution to confirm that the laccase present on each batch of ceCPTL had been effectively immobilized.

### 2.2. Morphology of Chitosan/Polyvinyl Alcohol/Tetraethyl Orthosilicate Tetraethylorthosilicate/Polypropylene/Glutaraldehyde/Laccase (ceCPTL)

After freeze drying, ceCPTL was lacerated into small pieces. The dried specimens were mounted on aluminum studs and coated with a gold–palladium alloy under high vacuum conditions for 90 s. The surface microstructures of the specimens were then examined using an ultra-high-resolution field-emission scanning electron microscope (NNS 230, Field Electron and Ion Company, Oregon City, OR, USA) operated at 10 kV.

### 2.3. Coloration

Each 1-cm^2^ piece of ceCPTL with varying amounts of immobilized laccase (8–25 μg/cm^2^) was immersed in 1 mL of 20 mM guaiacol solution to obtain the laccase TTI prototype and to investigate ceCPTL coloration.

#### 2.3.1. Color Measurement

The color of the laccase TTI prototype was measured using six replicates with a color measurement spectrophotometer (Diffuse/8° Spectrophotometer, Hunter Associates Laboratory, Reston, VA, USA). The results are expressed in accordance with the CIELAB system. The parameters determined were the degrees of lightness (L*), redness (+a*) or greenness (−a*), and yellowness (+b*) or blueness (−b*). The difference in the coloration (Δ*E*) between the sample color and the darkest color of the laccase TTI prototype (the darkest color is indicated by the subscript 0) was calculated as follows:

(1) $$\Delta E=\sqrt{{\left(L^{*}-L_{0}^{*}\right)}^{2}+{\left(a^{*}-a_{0}^{*}\right)}^{2}+{\left(b^{*}-b_{0}^{*}\right)}^{2}}$$

#### 2.3.2. Absorbance of Coloration

Because the spectrophotometer could determine only the color of a single sample in each measurement, conducting numerous experiments could have caused time differences and interfered with the coloration data. Therefore, an enzyme-linked immunosorbent assay reader (BioTEK Instruments, Winooski, VT, USA) was used to detect the coloration of multiple samples simultaneously. Following the method of Jhuang et al. [7], guaiacol oxidation was measured on the basis of the increase in absorbance at 470 nm. Because the darkest color in TTI coloration was observed at an optical density (OD)_470_ of 3.50, the corresponding L*a*b* of the absorbance value was used to calculate Δ*E.* The coloration endpoint was defined as the minimum time taken for *ΔE* to become less than 5 even when the reaction time was extended [18]. The normalized absorbance [norm(Abs)] was then calculated as the OD_470_ of the sample divided by 3.50.

### 2.4. Kinetic Evaluation

Laccase TTI prototypes and LAB-inoculated milk were incubated at 4, 15, 25, and 37 °C. The coloration rate was calculated as the change in OD_470_ within a certain time interval. For calculating activation energies (*Ea*, kJ/mol) according to the Arrhenius expression, the color responses of TTIs and milk quality were analyzed as follows [3]:

*y* = *kt* + *y*_0_ (2)

ln *y* = ln *y*_0_ + *kt* (3)

where *y* is the OD_470_ or LAB count determined after incubation time *t*, and *k* is the kinetic constant of the reaction rate. Equations (2) and (3) were used to evaluate the zero-order and first-order reactions, respectively, and were integrated to obtain the reaction rate constant. *Ea* was then calculated as follows:

(4) lnk=−Ea/RT + lnA

where *R* is the general gas constant (8.314 J/K·mol), *T* is the absolute temperature (K), and *A* is the prefactor. *Ea* was estimated from the slope of the Arrhenius plot and was determined at least thrice. The coefficient of determination (*R*^2^) was calculated for each test. The highest *R*^2^ was selected to represent the accuracy of *Ea* estimation.

### 2.5. Simulation of Milk Quality Change

LAB (*Lactococcus lactis* subsp. *lactis*) were purchased from the Bioresource Collection and Research Center (BCRC 12312) of the Food Industry Research and Development Institute (Hsinchu, Taiwan). Following BCRC instructions, LAB were cultured in a brain–heart infusion at 37 °C for 16 h to obtain a stationary phase (approximately 10^9^ colony-forming units (CFU)/mL) for use in milk quality tests. For the study purpose, samples with identical quality were used rather than practically meaningful samples. Therefore, by following the method of Haugen et al. [19], 1 mL of 10^2^ CFU/mL LAB was inoculated into 9 mL of ultra-high-temperature milk (Kuang Chuan Dairy Co. Ltd., Taoyuan, Taiwan). Because temperatures lower than 10 °C are unsuitable for the growth of LAB [20], milk was incubated at 10, 15, 25, and 37 °C in the storage experiment to simulate the growth of LAB in an abnormal storage temperature environment. The changes in LAB growth, pH, and titratable acidity (TA) were determined using the methods of Penasa et al. [21] and were employed to indicate the extent of milk quality change that occurred during storage. Each determination was performed in triplicate at least.

### 2.6. Response of the Laccase Time–Temperature Indicator (TTI) Prototype and Lactic Acid Bacteria (LAB) Growth in Milk

#### 2.6.1. Isothermal Response Test

The LAB-inoculated milk and laccase TTI prototype were stored at isothermal temperatures of 10, 15, 25, and 37 °C. The prediction error was calculated in accordance with the approach of Tsironi et al. [22] to determine the predictive accuracy of the laccase TTI prototype in response to the growth of LAB in milk:

(5) $$\mathrm{Prediction}\;\mathrm{error}\;\left(\%\right)=\frac{t_{LAB}-t_{TTI}}{t_{LAB}}$$

where *t_LAB_* and *t_TTI_* represent the time taken for the LAB concentration to reach 10^6^ CFU/mL (h) and the coloration endpoint (h) of the laccase TTI, respectively.

#### 2.6.2. Dynamic Temperature Response Test

Following the method of Kim et al. [4], temperature fluctuations between 4 °C (refrigeration temperature) and 25 °C (room temperature) were applied to simulate dynamic storage conditions. Incubation of the LAB-inoculated milk and laccase TTI prototype at 4 °C for 8 h, followed by storage at 25 °C for 8 h, was performed three times. After each incubation of 8 h, the growth of LAB in the milk and OD_470_ of the TTI prototype were quickly determined, and the LAB-inoculated milk and TTI prototype were then incubated again at the other temperature.

### 2.7. Statistical Analysis

All treatments were performed in triplicate at least. All statistical analyses were conducted in triplicate, and the results are represented as the mean and standard deviation. When the results of analysis of variance indicated significance (*p* < 0.05), data means were compared using the least significant difference test by employing SPSS (SPSS Inc., Chicago, IL, USA).

## 3. Results and Discussion

### 3.1. Immobilized Laccase on Electrospun Chitosan (CS) Fibers

**Figure 1:**
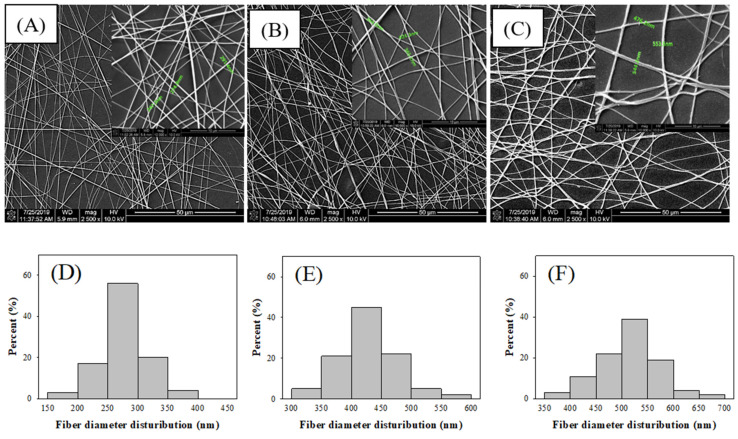
Scanning electron microscopy images (inset: 10,000× images) and the fibers diameter distribution of (**A**,**D**) chitosan/polyvinyl alcohol/tetraethyl orthosilicate tetraethylorthosilicate/polypropylene (CS/PVA/TEOS/PP), (**B**,**E**) CS/PVA/TEOS/PP/glutaraldehyde (GA), and (**C**,**F**) ceCPTL (CS/PVA/TEOS/PP/GA/laccase) with 25 μg/cm^2^ laccase immobilized, respectively.

PVA could be used as a cooperator to enhance the mechanical property of the electrospun CS fibers through the formation of intermolecular and intramolecular hydrogen bonds between the CS and PVA side chains. A polymer blend of PVA with CS was selected as the organic polymer and was cross-linked with TEOS through the coupling reaction [15], with the sol-gel method used to prepare hybrid fibers. The morphologies of electrospun CS/PVA/TEOS fibers with diameters mainly distributed in 250–300 nm were observed (Figure 1A,D). Large fibers of diameter mainly distributed in 400–450 nm, which had a smooth appearance, were observed after the fibers were soaked in GA (Figure 1B,E). Moreover, the ceCPTL fibers onto which 25 μg/cm^2^ laccase was immobilized were considerably thicker (mainly distributed in 500–550 nm) than the fibers without laccase immobilization (Figure 1C,F).

Susanto et al. [14] reported that cross-linking PVA/CS nanofibers with GA resulted in morphological changes to the membrane microstructure, such as an increase in fiber diameter. The addition of GA may produce a more stable membrane because, theoretically, it more efficiently cross-links the free –OH groups of PVA and amine groups of CS to the aldehyde group of GA. Koloti et al. [23] reported that laccase enzymes could be anchored on fibers through the formation of bonds with the abundant peripheral amine groups of CS by using GA as a cross-linker, which resulted in the increased thickness of the laccase-modified membrane surface; this was attributed to the swelling of nanofibers onto which the enzymes were covalently immobilized. In the present study, the diameters of the CS/PVA/TEOS fibers were higher after the fibers had been soaked in GA because the attached GA expanded the molecular chains, and laccase immobilization on the swollen fibers also increased the width of the ceCPTL fibers.

### 3.2. Coloration of the Laccase TTI Prototype

**Figure 2:**
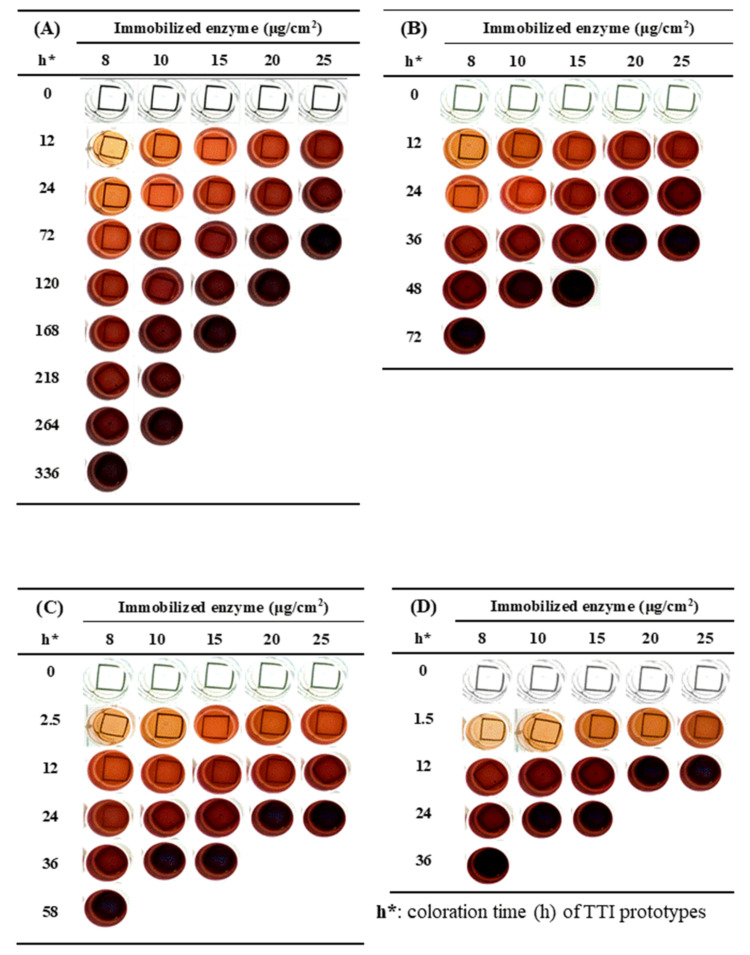
Color change of laccase time–temperature indicator (TTI) prototypes onto which various amounts of laccase (8–25 μg/cm^2^ on ceCPTL) were immobilized under storage at (**A**) 4, (**B**) 15, (**C**) 25, and (**D**) 37 °C.

The ceCPTL fibers onto which various amounts of laccase were immobilized were immersed in the guaiacol solution at 4 °C, and the color of laccase TTI prototype changed from transparent to light brown, dark brown, reddish brown, reddish purple, and finally purplish brown; the color thus gradually deepened during isothermal coloration (Figure 2). Through comparison of Δ*E* (between the sample after coloration and the darkest color of the laccase TTI prototype reacted for 202 h at 25 °C) with OD_470_, Δ*E* was discovered to be less than 5 when OD_470_ was 3.04 ± 0.06 (Table 1) and such reaction time was regarded to be the coloration endpoint at which the color change of the laccase TTI prototype could not be distinguished visually [18]. Norm(Abs) was approximately 0.87 when the laccase TTI prototypes reached the coloration endpoint.

At a given temperature, the coloration rate increased with an increase in the amount of immobilized laccase; for the same amount of immobilized laccase, the coloration rate increased with an increase in the storage temperature (Figure 2). These results revealed that the coloration response duration of the laccase TTI prototype can be regulated through adjustment of the amount of immobilized laccase.

### 3.3. Arrhenius Activation Energy (Ea) of Laccase TTI Coloration and LAB Growth in Milk

Variation in the initial bacterial count of a sample had to be avoided, especially when the initial bacterial count was zero, because the growth of bacteria as a spoilage indicator would otherwise lack credibility. According to Yang [17], LAB are the quality indicator bacteria of refrigerated pasteurized milk in Taiwan. Therefore, the effect of storage conditions on microbial growth was evaluated by adding LAB to milk.

**Figure 3:**
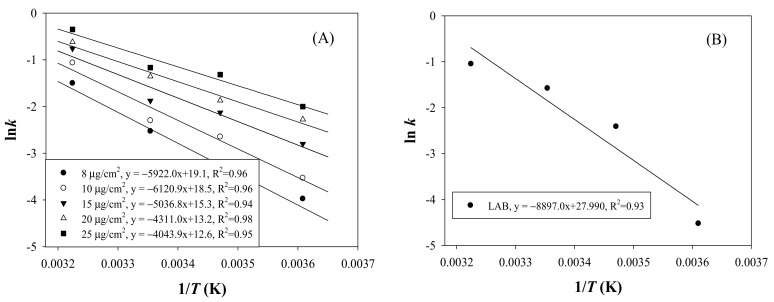
Arrhenius plots of ln *k* versus 1/*T* for (**A**) coloration of the TTI prototypes onto which various amounts of laccase were immobilized and (**B**) lactic acid bacteria (LAB) growth in milk.

To accurately predict LAB growth in milk by using the laccase TTI prototype, the kinetics of the coloration of the laccase TTI prototype and LAB growth in milk were analyzed. Accordingly, ceCPTL specimens onto which 8, 10, 15, 20, and 25 μg/cm^2^ laccase were immobilized were placed in the guaiacol solution, and the coloration test was performed at 4, 15, 25, and 37 °C. Equations (2) and (3) were used to evaluate the zero-order and first-order reactions, respectively. The results indicated a strong linear relationship between the reaction rate constant (ln *k*) and temperature (1/*T*) when the first-order reaction was considered. All determination coefficients (*R*^2^) were >0.94 for the regression lines of TTI prototypes with the laccase amounts 8–25 μg/cm^2^ (Figure 3A).

**Figure 4:**
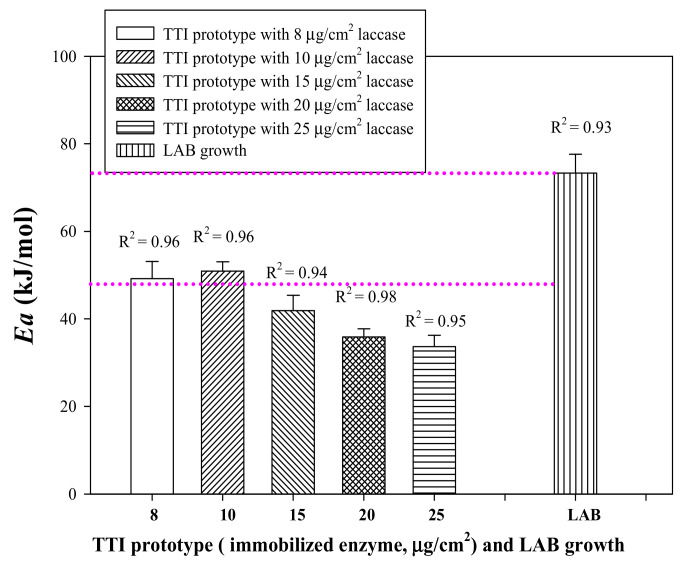
Arrhenius activation energy (*Ea*) of laccase TTI prototype coloration and LAB growth.

An integral method dependent on the relationship of 1/*T* with ln*k* was employed to evaluate the Arrhenius activation energy (*Ea)* of the coloration of the laccase TTI prototype. The *Ea* of the laccase TTI prototype onto which 8–25 μg/cm^2^ laccase was immobilized (i.e., 0.0086–0.0268 U) was 33.62 ± 2.62 to 50.89 ± 2.16 kJ/mol, and the corresponding *R*^2^ was >0.94 (Figure 4). However, Kim et al. [5] found that *Ea* varied only in the range of 43.90–45.44 kJ/mol for the free laccase concentration of 0.104–0.650 U/mL. The reason for the discrepancy in the *Ea* of these two types of laccase TTI was the immobilization of laccase on electrospun fibers with a high specific surface area in the present study. Jhuang et al. [7] reported that the Brunauer–Emmett–Teller (BET) specific surface area of their electrospun CS/PVA fibers was approximately 17.0455 m^2^/g, which was approximately 247 times of that of the non-electrospun film (20 mL of CS/PVA gel was spread evenly on a 10 × 20-cm^2^ PP film). Electrospun submicron fibers with a high BET specific surface area have a large and effective immobilization area that results in large gaps between laccase immobilized on the fibers [12]. This greater space results in fewer spatial structural obstacles and thus preserves the structural integrity of the immobilized enzymes for contact and reaction with reactants [13]. Therefore, the desirable coloration and *Ea* change could be obtained at a small amount of laccase in this study.

The first-order reaction is the most commonly used model to describe the deterioration of a quality attribute or the degradation of a nutrient in foods [24]. During the milk storage test, the LAB count was input to Equation (3), the first-order reaction equation. Linear regression indicated a strong correlation between the reaction rate constant and temperature (*R*^2^ = 0.93) (Figure 3B). This result revealed that the change in the LAB count during milk storage, when used as a quality indicator, exhibited a linear relationship described by a first-order reaction; this finding is consistent with the milk quality monitoring results obtained by Kim et al. [25]. Thus, using the equation of the first-order reaction, the *Ea* of LAB growth was calculated to be 73.32 ± 4.28 kJ/mol (Figure 4).

According to Kim et al. [4], the allowable difference in *Ea* between foods and TTIs is ± 25 kJ/mol for predicting food quality, with an error of <15%. The laccase TTI prototype coloration and LAB growth had an allowable *Ea* difference of <25 kJ/mol, within the range between the dotted line in Figure 4, when the amount of immobilized laccase was 8 (49.20 ± 3.91 kJ/mol) and 10 (50.89 ± 2.16 kJ/mol) μg/cm^2^. Therefore, the TTI prototype onto which 8–10 μg/cm^2^ laccase was immobilized was selected for testing the isothermal storage temperature for determining the effectiveness of the laccase TTI prototype under temperature abuse conditions other than the temperature of 4 °C, thus providing information regarding milk quality changes. The effects of long-term storage at non-refrigeration temperatures (10, 15, 25, and 37 °C isothermal storage temperatures) and of fluctuating temperatures (a 4 and 25 °C dynamic temperature cycle) on LAB growth in milk were then simulated, and the consequent coloration of the laccase TTI prototype was investigated.

### 3.4. Milk Quality Change during Storage

**Figure 5:**
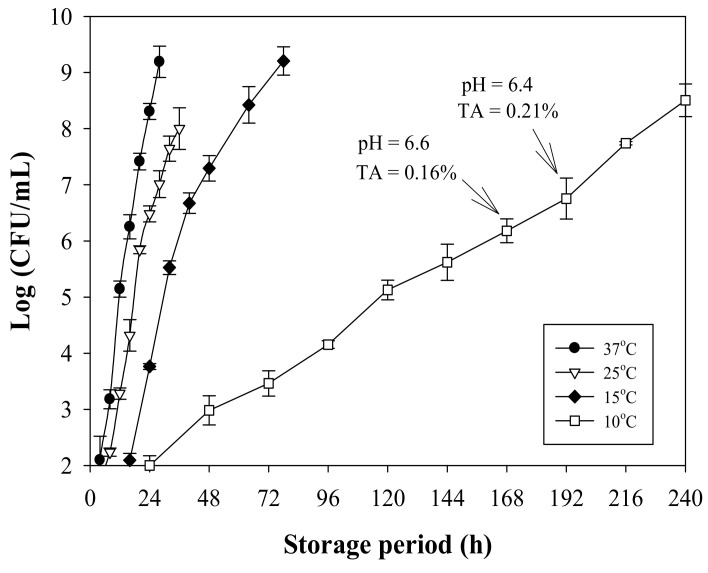
LAB growth and quality change in milk stored at various temperatures.

LAB growth increased markedly after the hysteresis period when the milk was stored at temperature >10 °C (Figure 5). Although the bacterial count was not the only criterion for judging milk deterioration, milk was considered to have expired when the LAB count reached 5–6 log CFU/mL [26]. In a dairy experiment with bacteria addition, unacceptable milk quality was indicated by a cell count of >6 log CFU/mL [27,28]. Microbial growth remains a crucial indicator when evaluating milk quality.

The pH of commercially available milk is 6.6–6.8, and the TA of pasteurized milk is approximately 0.16–0.18% [28,29]. The bacteria had already induced a quality change (LAB count > 10^6^ CFU/mL) when the pH began to decrease and the TA began to increase remarkably (Figure 5). Therefore, this study regarded the LAB count > 10^6^ CFU/mL as the index of quality change for milk stored at a temperature higher than the refrigeration temperature.

### 3.5. Response of Laccase TTI Prototype Coloration to Milk Quality Change

In the isothermal response test of milk containing LAB, 167.8 ± 1.5, 43.2 ± 2.3, 23.7 ± 1.7, and 15.6 ± 2.0 h were required for the LAB count to reach 10^6^ CFU/mL in the milk stored at 10, 15, 25, and 37 °C, respectively. The norm(Abs) of coloration for laccase TTI prototypes with 10 μg/cm^2^ immobilized laccase was approximately 0.87, and the prediction error was small (<10%) at every temperature (Table 2). According to Lin et al. [29], the TTI could accurately and effectively monitor the quality change when the TTI color reaction endpoint time was applied to predict the critical food quality deterioration time (actual shelf life), and the prediction error was <15%. Moreover, a positive value of prediction error indicated that the TTI reached the coloration endpoint before quality deterioration. The results of this isothermal response test in this study revealed that the color response of the TTI prototype onto which 10 μg/cm^2^ laccase was immobilized was sufficient and desirable for predicting LAB growth in milk.

**Figure 6:**
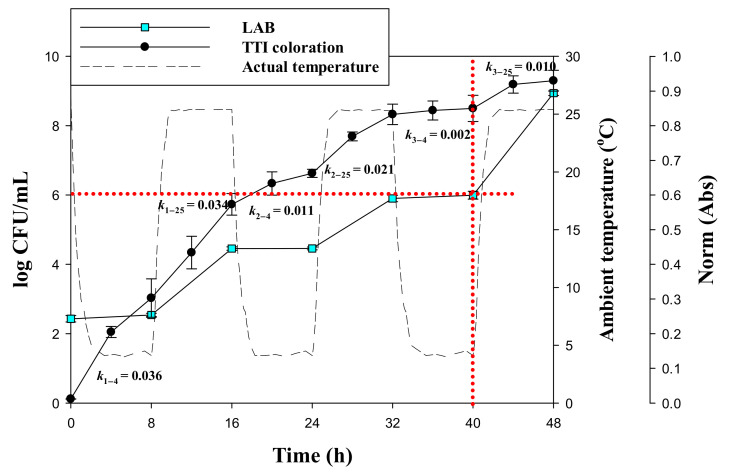
Correlation between coloration of the TTI prototype onto which 10 μg/cm^2^ laccase was immobilized [*k* is the coloration rate, norm(Abs)/h; the 1st and 2nd subscripts of *k* represent the number of cycles and temperature, respectively] and LAB growth in milk under dynamic temperature conditions; three cycles of 8 h each at 4 and 25 °C.

The kinetic parameters of the laccase TTI prototypes were further verified under non-isothermal conditions to simulate temperature fluctuations, which may occur in a cold chain. Therefore, to determine whether laccase TTI prototype coloration was reproducible under temperature fluctuations, it was necessary to investigate the increase in coloration with an increase in temperature and then the decrease in coloration with a decrease in temperature to verify the reliability of the obtained milk quality information. The TTI prototype with 10 μg/cm^2^ immobilized laccase and LAB-containing milk were employed in dynamic temperature response tests. The laccase TTI prototype and milk were incubated to simulate normal and abnormal storage temperatures 4 and 25 °C, and the temperature was switched every 8 h; 8 h of 4 °C followed by 8 h of 25 °C was regarded as one cycle, and three cycles were performed over 48 h. The results showed that the reaction rate (*k*) of laccase at 4 °C was lower than that at 25 °C in all cycles, except the first (Figure 6), which indicated that the laccase TTI prototype exhibited a favorable response to temperature, and that the coloration was reproducible under temperature fluctuations. Temperature sensitivity was thus retained by the immobilized laccase during temperature fluctuations, and dynamic modeling performed through kinetic parameters and numerical analysis was effective; the present findings are similar to the results of a reaction kinetics study of a lipolytic enzyme TTI examined under dynamic temperature profiles [30]. Because the coloration of the laccase TTI prototype was considered to follow a first-order reaction, the initial reaction occurred quickly. Thus, in the first cycle, the rate of TTI coloration at a 4 °C storage temperature [*k*_1–4_ = 0.036 norm(Abs)/h for 0–8 h] was slightly higher than that at 25 °C [*k*_1–25_ = 0.034 norm (Abs)/h for 8–16 h]. However, irrespective of temperature, the reaction rate in the first temperature cycle was higher than that in the third cycle, in which the coloration of the laccase TTI prototype was close to the endpoint and the rate of coloration decreased. TTIs are employed to determine whether a food product’s quality is unacceptable. Although the initial color reaction occurred quickly for the present TTI prototype, this did not affect the reliability of the quality change predicted using the TTI.

The dynamic temperature profiles showed that the LAB count in milk increased rapidly at 25 °C but then increased slowly at 4 °C, and this occurred for all three cycles. The LAB count in milk reached 10^6^ CFU/mL after 40 h of storage (Figure 6). At this time, the norm(Abs) of the laccase TTI prototype was approximately 0.87, which was close to the coloration endpoint. Neglecting the accumulated LAB count that increased slowly at 4 °C, the LAB count reached 10^6^ CFU/mL after 16 h when LAB growth at 25 °C was separately evaluated in the dynamic temperature test. However, the LAB count reached 10^6^ CFU/mL within 23.7 ± 1.7 h when the milk was isothermally stored at 25 °C (Figure 5). LAB were expected to grow continuously during temperature increase and decrease periods within the fluctuation cycle. Clearly, temperature fluctuations during storage considerably influence milk quality. If the milk storage environment deviates from the appropriate refrigeration temperature, the risk of product deterioration before or on the expiration date specified on the label is considerably higher. In this situation, laccase TTI can provide information about the temperature history to the observer in order to assess the quality of the milk product.

## 4. Conclusions

Cold chain temperature is not always maintained within the recommended range; therefore, developing a real-time and cost-effective monitoring system for perishable foods is vital. Immobilization of a small amount of laccase on a TTI produced an *Ea* and color response consistent with LAB growth in milk and provided visible information of the product temperature history. Managers and consumers can monitor milk quality by considering a TTI’s color changes along with the expiration date printed on labels. Because the enzyme in the TTI is immobilized on submicron electrospun fibers, the amount of laccase required is small, minimizing TTI production costs, which is beneficial in commercial applications.

## Figures and Tables

**Table 1:** Color response correlates with coloration of TTI prototype (onto which 8 μg/cm^2^ laccase was immobilized) at 25 °C.

Time (h)	L*	a*	b*	Δ*E*	OD_470_
0.00	101.50 ± 1.94 ^a^	0.04 ± 0.03 ^f^	0.11 ± 0.16 ^e^	65.67 ± 0.84 ^a^	0.033 ± 0.00
4.00	86.24 ± 1.76 ^b^	14.61 ± 0.24 ^de^	17.14 ± 2.02 ^a^	52.64 ± 1.25 ^b^	1.209 ± 0.07
6.00	67.48 ± 1.90 ^c^	21.65 ± 0.96 ^a^	12.93 ± 1.84 ^b^	34.75 ± 0.91 ^c^	1.909 ± 0.04
18.00	52.59 ± 1.02 ^d^	21.67 ± 1.42 ^a^	6.71 ± 1.84 ^c^	19.53 ± 0.96 ^d^	2.58 ± 0.09
22.00	49.63 ± 1.08 ^e^	20.54 ± 1.29 ^a^	4.25 ± 1.29 ^cd^	15.64 ± 0.64 ^e^	2.64 ± 0.09
26.00	45.73 ± 0.73 ^g^	18.84 ± 1.33 ^b^	2.17 ± 1.91 ^de^	10.60 ± 1.75 ^f^	2.81 ± 0.02
58.00	43.37 ± 1.07 ^gh^	18.08 ± 1.45 ^bc^	0.42 ± 1.55 ^de^	4.55 ± 1.52 ^g^	3.04 ± 0.06
82.00	41.47 ± 1.05 ^hi^	16.43 ± 0.97 ^cd^	−0.78 ± 1.51 ^e^	3.58 ± 0.93 ^g^	3.19 ± 0.08
106.00	40.24 ± 1.26 ^hi^	15.56 ± 1.65 ^d^	−0.84 ± 1.46 ^e^	3.47 ± 0.88 ^g^	3.27 ± 0.04
202.00	37.24 ± 1.03 ^j^	13.38 ± 1.17 ^d^	−2.06 ± 1.16 ^e^	0.00 ± 0.87 ^h^	3.50 ± 0.00

a–j The different superscript for sample in each column represents significant difference (*p* < 0.05).

**Table 2:** Prediction error of coloration of laccase TTI prototype for monitoring LAB growth in milk.

	Immobilized Enzyme (μg/cm^2^)	Coloration Endpoint (h)		Prediction Error (%)		Time for LAB Count Reached 10^6^ CFU/mL (h) ^1^
Temperature (°C)		8	10	8	10	
10		190.8 ± 4.9	165.6 ± 3.7	−13.7	1.3	167.8 ± 2.5
15		57.9 ± 2.1	39.5 ± 3.4	−34.0	8.6	43.2 ± 2.3
25		34.1 ± 2.6	21.8 ± 1.5	−43.9	8.0	23.7 ± 1.7
37		27.5 ± 3.3	15.2 ± 2.4	−76.2	2.6	15.6 ± 2.0

^1^ Time for LAB count reached 10^6^ colony-forming units (CFU)/mL (h) was estimated with the regression of LAB growth curve.

## Data Availability

Data are available on request to the corresponding author.
